# Efficacy and safety of mechanical thrombectomy for cardioembolic stroke: A protocol for systematic review and meta-analysis

**DOI:** 10.1097/MD.0000000000024340

**Published:** 2021-01-15

**Authors:** Ziqu Zhang, Chenjin Wang, Wengang Xia, Jingwei Li, Yali Wang, Yong Liu

**Affiliations:** a Chengdu University of Traditional Chinese Medicine, Chengdu; b Department of Cardiology, Zigong Third People's Hospital, Zigong, Sichuan, China.

**Keywords:** cardioembolic stroke, mechanical thrombectomy, meta analysis, protocol, systematic review

## Abstract

**Background:**

Several randomized clinical trials have demonstrated the safety and efficiency of mechanical thrombectomy in the management of acute ischaemic stroke caused by larger vessel occlusion. According to the trial of Org 10172 in Acute Stroke Treatment (TOAST) classification, acute ischaemic stroke can be divided into cardioembolic stroke and non-cardioembolic stroke. Previous studies have shown that mechanical thrombectomy in cardioembolic stroke with intracranial large artery occlusion has a poor prognosis. The reason may be that the old emboli are hard, making it difficult to remove. However, recent evidence shows that mechanical thrombectomy is also effective and safe in patients with cardioembolic stroke. Therefore, the aim of this study is to evaluate the efficacy and safety of mechanical thrombectomy for cardioembolic stroke.

**Methods:**

The electronic database, including PubMed, Cochrane Library, EMBASE, the China National Knowledge Infrastructure (CNKI), China Biology Medicine disc (CBM), VIP database, and Wan-fang database, were thoroughly retrieved from inception to December 1, 2021, without language restrictions. All randomized controlled trials that evaluated the efficacy and safety of mechanical thrombectomy in the treatment of cardioembolic stroke will be included. Primary outcomes will include vascular recanalization rate and score scale. Two authors will independently scan the articles searched, extract the data from articles included, and assess the risk of bias by Cochrane tool of risk of bias. Disagreements will be resolved by discussion among authors. All analysis will be performed based on the Cochrane Handbook for Systematic Reviews of Interventions. Dichotomous variables will be reported as risk ratio or odds ratio with 95% confidence intervals and continuous variables will be summarized as mean difference or standard mean difference with 95% confidence intervals.

**Results:**

This review will be to assess the efficacy and safety of mechanical thrombectomy for cardioembolic stroke.

**Conclusions:**

The results of our findings may be helpful for clinicians and health professionals to re-examine the clinical decision-making in the treatment of cardioembolic stroke, promising way for treatment of patients with cardioembolic stroke.

**Systematic review registration number:**

INPLASY2020120035

## 1 Introduction

Stroke, a major cause of serious disability for adults, is the second-leading cause of death in the world.^[1]^ Two main stroke types are known as hemorrhagic stroke and ischemic stroke.^[2]^ The commoner type is an ischemic stroke, caused by interruption of blood flow to a certain area of the brain, accounting for 85% of all acute strokes.^[3]^ Cardioembolic stroke (CES), a type of ischemic stroke, according to the trial of Org 10172 in Acute Stroke Treatment (TOAST) classification, is often severe because it is characterized by larger lesions than other types of stroke, and its recurrence rate is higher than that of other stroke types.^[3,4]^ Approximately 25% of all ischemic cases are believed to be cardioembolic in origin.^[5]^ And as the population ages, the incidence of CES is anticipated to rise.^[6]^ The importance of treatment of patients with CES is significant due to the burden of disease and the severity of the illness.

Several randomized clinical trials have demonstrated the safety and efficiency of mechanical thrombectomy (MT) in the management of acute ischaemic stroke.^[7–12]^ Patients with a large vessel occlusion in the anterior circulation also benefit from MT following intravenous thrombolysis with tissue plasminogen activator.^[13,14]^ However, the benefits of MT in the treatment of CES are still under discussion. Previous studies have shown that MT in CES with intracranial large vessel occlusion has a poor prognosis.^[15]^ The reason may be that the old emboli are hard, making it difficult to remove.^[15,16]^ But recent studies have shown that patients with CES can benefit from MT, not only in terms of efficacy, but also in safety.^[17]^ Therefore, our goal is to conduct a meta-analysis of published studies to evaluate the efficacy and safety of MT for CES.

## 2 Methods and analysis

### 2.1 Objectives and registration

This review will be to evaluate the efficacy and safety of mechanical thrombectomy for cardioembolic stroke. This review protocol is registered with the International Platform of Registered Systematic Review and Meta-Analysis Protocols (INPLASY) on December 6, 2020, and was last updated on December 6, 2020, (registration number INPLASY2020120035). Additionally, this review will adhere to the preferred reporting items for systematic reviews and meta-analyses statement.^[18]^

### 2.2 Eligibility criteria

#### 2.2.1 Types of studies

Randomized controlled trials (RCTs) will be included in this systematic review regardless of publication status and language. Quasi-randomized controlled trials (QRCTs) and nonrandomized studies will be excluded.

#### 2.2.2 Types of patients

Patients include those diagnosed with cardiogenic stroke, regardless of gender, age, nationality, or race.

#### 2.2.3 Patient and public involvement

In this study, there is no patient and public involvement in consideration of this protocol for a systematic review.

#### 2.2.4 Types of interventions

The treatment group is treated with mechanical thrombectomy, or mechanical thrombectomy combine with other conventional treatments. The control group were patients with cardiogenic stroke who were treated with intravenous thrombolysis or intravascular interventional therapy other than mechanical thrombectomy.

### 2.3 Types of outcome measures

The main outcome measures we sought at the vascular recanalization rate, the rate of incidence of adverse events, the rate of mortality within 90 days, preoperative and postoperative 7 days NIH Stroke Scale score,^[19,20]^ postoperative 90 days Modified Rankin Scale score.^[21]^

### 2.4 Search methods for the identification of studies

#### 2.4.1 Electronic searches

We will use computers to retrieve all RCTs of mechanical thrombectomy on PubMed, Cochrane Library, EMBASE, CNKI, Wan-fang, CBM and VIP databases.

#### 2.4.2 Searching other resources

We will also have manual retrieval for relevant conference reports, and contact experts in the field and corresponding authors to obtain important information that cannot be obtained by the above retrieval.

### 2.5 Data collection and analysis

#### 2.5.1 Selection of studies

**Figure 1:**
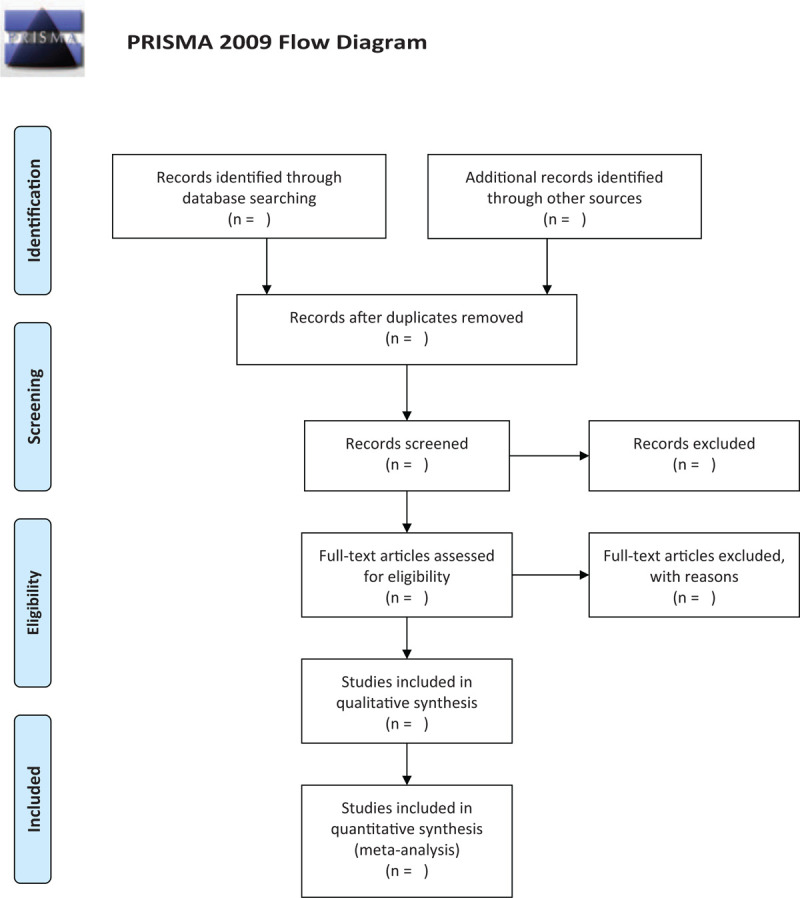
Flow diagram of studies identified.

To determine the studies to be searched further, 2 review authors (ZZ and CW) will independently scan the titles and abstracts of all articles identified from electronic databases. Full-text articles will be scanned for all potentially relevant articles. If there is any disagreement on the selection of articles, they will be discussed with the 3rd author (WX). The selection process will be shown in a preferred reporting items for systematic review and meta-analysis (PRISMA) flow chart (Fig. 1).

#### 2.5.2 Data extraction and management

For all studies included, 2 review authors (ZZ and CW) will independently extract the relevant information using a standard data extraction table. Information will include publication of year, author, participants, intervention, control, duration of intervention, outcomes, and methodologic characteristics. Disagreements will be resolved by discussion by arbiter (WX).

#### 2.5.3 Assessment of the risk of bias in the included studies

To assess the risk of bias for all included studies, Cochrane Collaboration's bias risk tool will be used by 2 independent review authors (ZZ and CW) to assess the following areas: random sequence generation, allocation concealment, blindness to participants, people, and results, incomplete outcome data, optional reporting, and other biases. Any discrepancies in the deviation risk assessment will be resolved through discussion. Ultimately, the quality of the studies will be divided into 3 levels: low risk of bias, high risk of bias, and unclear risk of bias. Disagreements will be resolved by discussion by arbiter (WX).

#### 2.5.4 Measurement of the treatment effect

##### 2.5.4.1 Strategy of data synthesis

All analyses will be conducted by using RevMan software (V5.4), we will select fixed effects model or random effects model to merge the outcome indicators in accordance with the results of heterogeneity test. The fixed effects model will be applied for data synthesis of low heterogeneity (I^2^ < 50%) while the random effects model will be conducted if the heterogeneity is significant (I^2^ ≥ 50%). It is considered that differences are statistically significant if the results of Z test show that *P* value is less than .05, and the 95% confidence intervals does not contain 0 (for continuous variables) or the 95% confidence intervals does not contain 1 (for dichotomous variables).

#### 2.5.5 Dealing with missing data

For insufficient or missing data, we will contact the authors by e-mail or phone as much as possible. All analysis will be performed based on intent-to-treat principle.

#### 2.5.6 Assessment of heterogeneity

Heterogeneity will be identified by visual inspection of the forest and tested by standard Chi-squared statistic and a significance level of 0.1. Additionally, the I^2^ statistic will be used to examine heterogeneity for quantifying inconsistency in the included studies. Fixed or random effects models will be performed in meta-analysis. If I^2^ >0.5, random effects models will be used.^[22]^

#### 2.5.7 Assessment of reporting biases

Funnel plots will be used to assess the potential for small study bias if there are sufficient studies. Asymmetry of funnel plots will suggest possible small study effects and the results will be explained cautiously.^[23,24]^

#### 2.5.8 Data synthesis

Meta-analysis will be performed using RevMan 5.4 software. When there is no statistical heterogeneity among the results, a fixed-effects model will be used for meta-analysis. When there is statistical heterogeneity among the results, the heterogeneity source will be further analyzed and a random-effects model will be used for meta-analysis after excluding the effects of significant clinical heterogeneity. When there is significant clinical heterogeneity, we will use subgroup analysis or sensitivity analysis, or only descriptive analysis.

#### 2.5.9 Subgroup analysis

We will perform the following subgroup analyses if included data are highly heterogeneous: by age, sex, sample size, type of interventional stents, highly heterogeneous.

#### 2.5.10 Sensitivity analysis

Sensitivity analysis is an important method primarily used to assess the robustness and reliability of the combined results of meta-analysis. It is a commonly used sensitivity analysis method to combine the effect size after eliminating each of the included studies, or after changing the inclusion or exclusion criteria or eliminating certain types of studies. For possible low-quality studies, sensitivity analysis is required. If the heterogeneity of the included literature is significant, in order to ensure the credibility of the research results, we will conduct sensitivity analysis by excluding each included study separately, so as to improve the research quality.

#### 2.5.11 Ethics and dissemination

This systematic review and meta-analysis will not require ethical approval because there are no data used in our study that are linked to individual patient data. In addition, findings will be disseminated through conference presentations and peer-review publications.

#### 2.5.12 Confidence in cumulative evidence

In this study, the level of evidence on outcomes will be assessed using an approach based on the grading of recommendations assessment, development and evaluation (GRADE). The quality of the body of evidence will be assessed based on 5 factors, including study limitations, effect consistency, imprecision, indirectness, and publication bias. The assessments will be categorized as high, moderate, low, and very low quality.

## 3 Discussion

CES has caused a heavy economic burden. The recurrence rate of this type of stroke is about 12% at 3 months, higher than that of non-cardioembolic stroke. The severity of cardioembolic strokes and the resulting disability are greater than with non-cardioembolic stroke.^[25]^ It is of great significance for the effective and safe treatment of CES. Mechanical thrombectomy, as a rising means of treatment in recent years, has been widely recognized in the treatment of ischemic stroke caused by large vessel occlusion.^[26]^ The efficacy and safety of MT in the treatment of CES are also worth studying. It is therefore necessary to systematically assess the efficacy and safety of MT in treating patients with CES. The results of our findings may be helpful for clinicians and health professionals to re-examine the clinical decision-making in the treatment of CES, promising way for treatment of patients with CES.

However, there are some potential limitations to this study. For example, difference in the length of occurrence time of ischemic stroke in patients and differences in the types of MT stents may result in significant heterogeneity. In addition, limited to language ability, we only search for documents in English and Chinese, and may ignore studies or reports in other languages.

## Author contributions

ZZ, CW and WX developed the study protocol. ZZ and CW developed the search strategy. ZZ and CW will scan the included studies, extract the data and assess the risk of bias. WX will act as an arbiter if there is any disagreement in this study. ZZ and CW will perform data analysis with supervision of WX and YL. All authors (ZZ, CW, WX, JL, YW and YL) will contribute to data interpretation. ZZ, CW and WX drafted and revised the manuscript. All authors have read and approved the final version of the manuscript.

**Conceptualization:** Ziqu Zhang, Chenjin Wang, Wengang Xia, Yong Liu.

**Data curation:** Ziqu Zhang, Chenjin Wang.

**Formal analysis:** Ziqu Zhang, Chenjin Wang, Wengang Xia.

**Methodology:** Wengang Xia, Yong Liu.

**Software:** Jingwei Li, Yali Wang.

**Supervision:** Wengang Xia, Yong Liu.

**Supervision:** Ziqu Zhang, Chenjin Wang, Wengang Xia, Yong Liu.

**Writing – original draft:** Ziqu Zhang, Chenjin Wang.

**Writing – review & editing:** Jingwei Li, Yali Wang, Wengang Xia.

## Abbreviations

Abbreviations: CES = cardioembolic stroke, CI = confidence intervals, MT = mechanical thrombectomy.
